# Cases of Tubercular Disease

**Published:** 1842-02-19

**Authors:** Brodie S. Herndon

**Affiliations:** Fredericksburg, (Va.)


*Cases of Tubercular Disease.* By Brodie S. Herndon, M. D.

Kitty, a coloured servant of the better class, accustomed to comforts, aged 30; hysterical; married to a man in robust health, though intemperate; the mother of six apparently healthy children, from twelve years to twelve months of age; lived in a good-sized, airy, and well-lighted room, though in a damp situation, and made use of a large ten-plate stove for cooking; the family living and sleeping in the same room.

In the summer of 1837, the oldest child was put out; in the fall of the same year the remaining five children were seized successively with cough, fever, disordered bowels, and wasting, and, eventually, all died within six months. In three of the cases, a post mortem examination revealed the most extensive tubercular deposits. The lungs presented the tubercular infiltration, with numerous abscesses; the bronchial glands, spleen, mesenteric glands, intestines, and liver were studded with tubercles; the brain was not examined. When three of the children had died, the remaining two were removed, with their mother, then pregnant, to another situation—a room warmed by a fire place—one of the children slightly complaining. In a short time, however, they sickened and died as the others had done. The mother, shortly after, gave birth to a healthy child, which continues well. The oldest child is also perfectly well.

Ophelia, sister of Kitty, mother of three children, and wife of a healthy man; also uses a stove for cooking in the same room in which the family sleep. Last summer one of her children, two years old, sickened and died, after protracted bowel complaint and wasting, supposed to be connected with teething. Body not examined. Another, aged four years, is now far advanced in disease, plainly the effect of tuberculous deposits in the chest and abdomen. The remaining child, an infant of ten months, seems well.

Both the women say their children were brought up in rooms warmed with stoves; and, until seisure, there were no healthier looking children. In most of the cases there were sudamina over the neck and chest. The opinion of M. Louis and others was fully borne out, that inflammation had nothing to do with the cases, except as a consequence. The immunity of the oldest child is doubtless the effect of avoidance of the causes attaching to the spot, and, in part, of the diminished susceptibility of her age—this being greatest, according to authors, about four or five years, and decreasing on to puberty, after which tubercular disease is nearly synonomous with phthisis. The order in which the organs were affected as to amount of disease, was the lungs, spleen, bronchial glands, mesenteric glands, intestines, and liver. The spleen was loaded with tubercles in all the cases. Four of Kitty's children were females, and the other a male. No treatment did them any good.

The report of the cases is imperfect, in many respects, the more so, because it is written from memory, after a considerable lapse of time, and because they were not observed with a view to publication. All the facts, however, may be entirely relied upon. A part of the present object in this communication is to avail myself of any suggestion you may make as to the case now on hand. Its progress and result can be carefully observed and reported.

*Fredericksburg*, (*Va.*) *Jan.*, 1842.

[The cases described by our correspondent are evidently of tuberculous disease, totally independent of local causes of inflammation. They are, therefore, strictly constitutional, and the only rational treatment is one of prevention, which is in itself difficult and doubtful. If the patients were in a situation in life to admit of entire change of residence and climate for some years, with rigid attention to hygienic means, such as exercise in the open air, and free exposure to the sun, there would be a reasonable probability of exemption from the disorder.

In a case of the kind alluded to, we consider change of residence of imperative necessity. If possible, the whole family should migrate to the south; but, at any rate, the room in which so many are confined should be changed for a more airy and dry one, and the children should have an abundance of nourishing food. Small portions of chalybeates might also be given with advantage for a long period, as soon as the age at which the tuberculous deposits generally develope themselves is reached. These doses should be minute; so as to produce no local irritation whatever; one or two drops of the ordinary solution of the hydriodate of iron, after some months, alternated for a time with a like quantity of Lugol's solution of iodine, seems to us a proper dose. In the West Indies, as well as the United States, it would seem that mulattoes are more subject to these general tubercular diseases, than the unmixed races.]

